# 

**Published:** 1899-03

**Authors:** 


### NOTICES.

### American Medical Association.

The next meeting of the American Medical Association will be held at Columbus, Ohio, June 6 to 9, 1899.

The Section on Stomatology will be of unusual interest, since its program will be unlike any ever presented at any other dental society meeting. It will be made up mostly if not entirely of original papers, although the papers presented before this section have always been of high order. Papers upon the manipulation and technique of operations and mechanical dentistry are never read at these meetings. Only such subjects as are of interest to the dental and medical professions are received. These papers have always been selected by the secretary with the greatest care. There is no business to transact in the section. Everything in this matter is referred to the business committee (composed of the last three retired Chairmen of Sections) which meets every afternoon at 5 P. M. to consider the welfare of the whole Association and of each section. These matters are discussed, and, if favorably considered, they are reported favorably to the general meeting. This method relieves the sections and general meetings of considerable routine work and also gives more time to do the regular business.

On Tuesday afternoon a committee of three is appointed in each section to nominate officers, a chairman and secretary, for the following year. The committee reports Wednesday, at which time the candidates are elected. Since the offices of chairman and secretary carry no great honor, there is very little desire on the part of the members for the position, hence politics never mar the session.

With the exception of a few years, the present secretary has been in office continuously since the section was established, eighteen years ago. There are many advantages in having a permanent secretary, since a new chairman is elected nearly every year a permanent secretary is familiar with the methods of conducting the section and knows just whom to call on for papers, no matter in what particular part of the country the Association may meet. The character of the papers are always under his supervision. With his general knowledge of the workings of the general meetings as well as the section, if he is interested, the work goes on without friction.

The meetings of the Section on Stomatology are never largely attended, principally for the reason that there are no attractions except the advancement of dental science. The smallest attendance at any one session of a meeting was twelve, the largest ninety-six, with an average of twenty-eight.

The members of the Section on Stomatology have never encouraged or desired merely large numbers. They have always felt that even ten or twelve who were good thinkers and talkers and of sufficient intelligence to discuss the various papers presented, was much more to be desired than a large number of uninterested people.

Dentists are admitted in the same manner as physicians. Dentists holding the D.D.S. degree, upon presenting credentials from their State or local society and the payment of $5, can become a members of the Association which also entitles them to the Journal of the Association for the coming year.

Any one who wishes to attend the sessions of the Section of Stomatology is permitted to do so.

The following list is the preliminary program of the meeting to date: Chairman's Address, Dr. G. V. I. Brown, Milwaukee; "Actinomycosis," Dr. Ludwig Hektoen, Chicago; "Evolution of Decay" (further experiments), Dr. A. C. Hart, San Francisco; "Cocaine and Eucaine, Their Relative Toxinity" (the results of original investigation and experimentation upon humans as well as animals), Dr. A. H. Peck, Chicago; "Epithelial Structures in the Peridental Membrane" (original investigation), Dr. Frederick Noyes, Chicago; "Infectious Ulcerative Stomatitis," Dr. John Marshall, Chicago; "Oral Surgical Operation" (with illustrations showing remarkable results), Dr. G. V. I. Brown, Milwaukee; "Some Points on the Etiology, Pathology and Treatment of Persistent Pyorrhœa Alveolaris," Dr. G. T. Carpenter, Chicago; "Interstitial Gingivitis" (so called Pyorrhœa Alveolaris), giving the result of original work with large photographic illustrations showing the progress of the disease from beginning to the loss of the teeth, Dr. Eugene S. Talbot, Chicago; "Syphilitic Infection from Dental Instruments, with Cases," Dr. W. L. Baum, Chicago; "Professional Education and Ethics," Dr. A. E. Baldwin, Chicago.

A revised and complete program will appear in the May number. G. V. I. Brown, *Chairman.*

Eugene S. Talbot, *Secretary.*

### Married.

At Rockwell, Texas, December 25th, 1898, Dr. Jessie Estelle Castle to Mr. Frank Lynn LaMoreaux.

Dr. Castle graduated from the dental department of the University of Michigan, at the end of the term 1896. Dr. Castle subsequently took advance work in the department from which she graduated, and very thoroughly equipped herself for the dental profession. After her graduation she practiced for a year or more in the Sanitarium at Battle Creek, Michigan, with very great acceptance. Dr. Castle-LaMoreaux will have the congratulations and best wishes of all those who knew her in the University, as she was a universal favorite. Her permanent home is 193 Home Street, Dallas, Texas.

May the fullness of happiness be hers throughout life's course, is the sentiment of the Register.

### Board of Dental Examiners of Pennsylvania.

Examination of applicants for license to practice dentistry in Pennsylvania will be held April 11th, 12th, 13th and 14th, in Philadelphia and Pittsburg, and June 13th, 14th, 15th and 16th, 1899, in Philadelphia. Applicants should state in which city they desire examination when they apply for examination blanks to the Dental Council, Harrisburg, Pa.

G. W. KLUMP, Sec'y,
Williamsport, Pa.

### THE DENTAL REGISTER,

A MONTHLY MAGAZINE DEVOTED TO THE INTERESTS OF THE

### DENTAL PROFESSION.

*Terms Reduced to* $2.00 *Per Year. Single Copies,* 25 *Cents.*

### EDITED BY PROF. J. TAFT, D.D.S.

—AND—

### Published by SAM'L A. CROCKER & CO., Ohio Dental and Surgical Depot.

The volume commences in January and closes in December.

The REGISTER being issued monthly is always fresh, therefore Indispensable to the practitioner who desires to keep posted up with the new inventions and improvements which are daily developed. Neither expense nor effort will be spared to give value and variety to its contents. Articles will be illustrated with well executed engravings whenever they will add to the interest or assist to explanation.

Its extensive circulation and frequency of issue make it one of the BEST DENTAL ADVERTISING MEDIUMS in the United States.

All subscriptions must begin with January or July.

Local or special notices, five lines or less, will be inserted for $3 each insertion; over five lines, 50 cts. per line.

All advertising bills payable in advance, or at furthest, after the issue of the first number containing the advertisement. All transient advertisements to be paid for in advance.

☞CONTRIBUTIONS SOLICITED.

Exclusively Editorial Communications should be addressed to the Editor.

The latest number of the REGISTER may be ordered with or without other goods at any time, and all letters should be addre[illegible]

